# Transthoracic echocardiographic imaging of coronary arteries: tips, traps, and pitfalls

**DOI:** 10.1186/1476-7120-6-7

**Published:** 2008-02-01

**Authors:** Fausto Rigo, Bruno Murer, Giovanni Ossena, Enrico Favaretto

**Affiliations:** 1 Department of Cardiology, Umberto I°Hospital, Mestre-Venice, Italy; 2 Department of Anatomic Pathology, Umberto I° Hospital, Mestre-Venice, Italy

## Abstract

The aim of this paper is to highlight coronary investigation by transthoracic Doppler evaluation. This application has recently been introduced into clinical practice and has received enthusiastic feedback in terms of coronary flow reserve evaluation on left anterior coronary artery disease diagnosis. Such diagnosis represents the most important clinical application but has in itself some limitations regarding anatomical and technological knowledge. The purpose of this paper is to offer a didactic approach on how to investigate the different segments of left anterior and posterior descending coronary arteries by transthoracic ultrasound using different anatomical key structures .as markers

We will conclude by underlining that, nowadays, innovative technology allows complete evaluation of both major coronary arteries in many patients in a resting condition as well as during pharmacology stress-tests, but we often do not know it.

## Coronary flow investigation by ultrasound: what can we get at present ?

The application of the latest ultrasound technology, in particular the 2^nd ^harmonic, has opened new roads in ultrasound coronary evaluation. By applying anatomical knowledge and the newest technical applications it is nowadays possible to propose a complete coronary evaluation of the left anterior descending artery and of a part of the posterior descending coronary artery in clinical practice [1] [see Additional File 1].

## Ultrasound coronary anatomy: reference points

**Figure 1:**
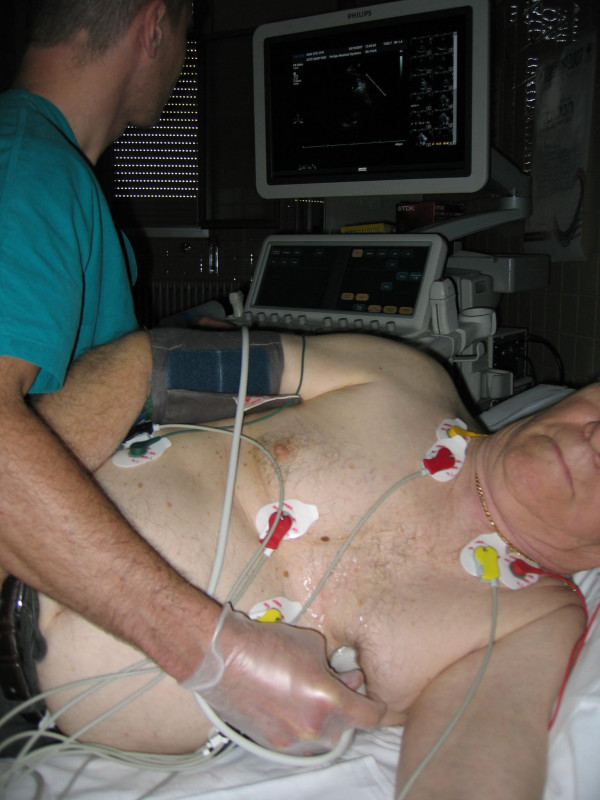
shows Transthoracic positioning of probe in order to highlight the two major coronary arteries while.

**Figure 2:**
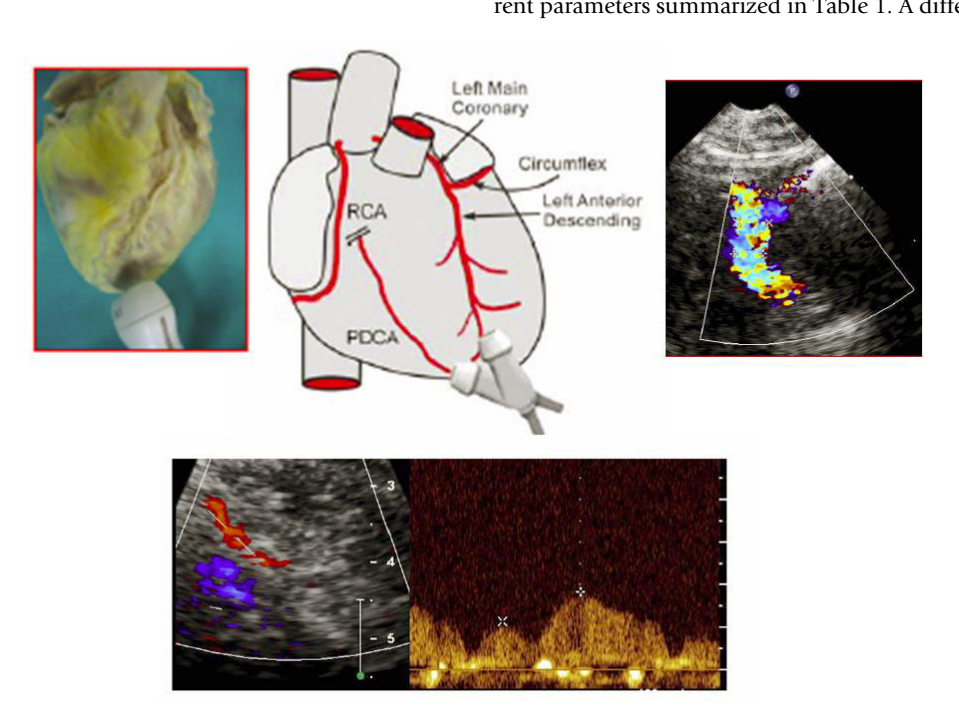
represents an artist's drawing illustrating transducer beam orientations to the left anterior descending coronary artery (LAD) and to posterior descending coronary arteries with the corresponding echocardiographic images of the mid-distal tract of LAD Pulse-wave flow and posterior descending coronary artery (PDCA).

Left anterior descending (LAD) coronary anatomy was the first artery investigated with ultrasound by transesophageal and transthoracic approach. This vessel is visible by using ultrasound from proximal to distal tract and following key anatomical structures through the delivery of an ultrasound beam in an off-axis approach starting from the classical apical approach (Fig 1, 2).

**Figure 3:**
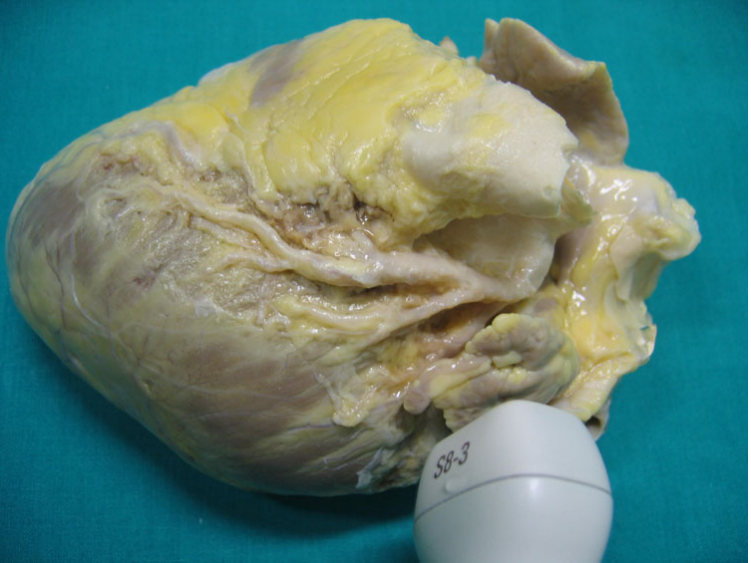
shows anatomical finding of proximal tract of Left anterior descending coronary artery (reference key are Aorta, Pulmonary artery and left atrial appendage).

**Figure 4:**
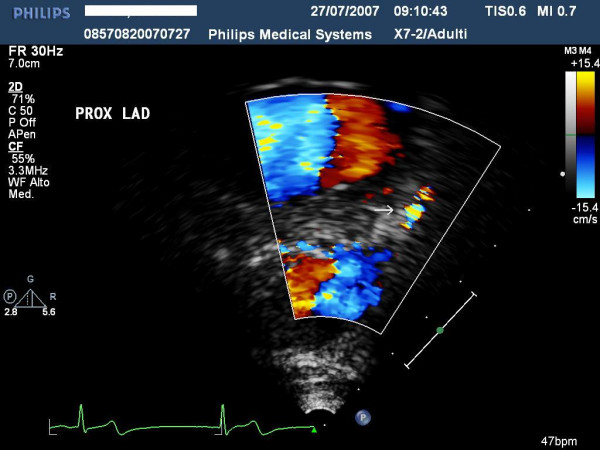
highlights the ultrasound findings of proximal left anterior highlighted with color-Doppler and.

**Figure 5:**
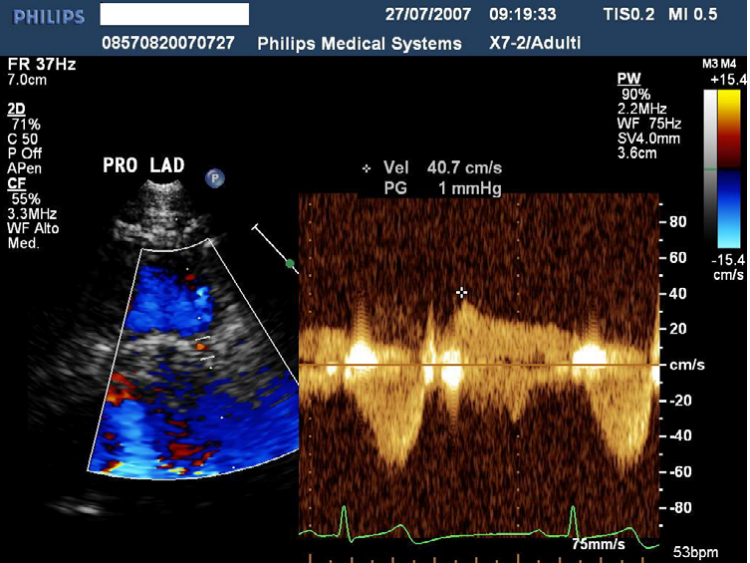
shows the pulse Doppler envelope of proximal tract of the left descending coronary artery.

Proximal Lad: the left atrial appendage and pulmonary artery represent the key reference points in detecting the proximal left anterior descending coronary tract (Fig 3, 4, 5) [see Additional File 2].

**Figure 6:**
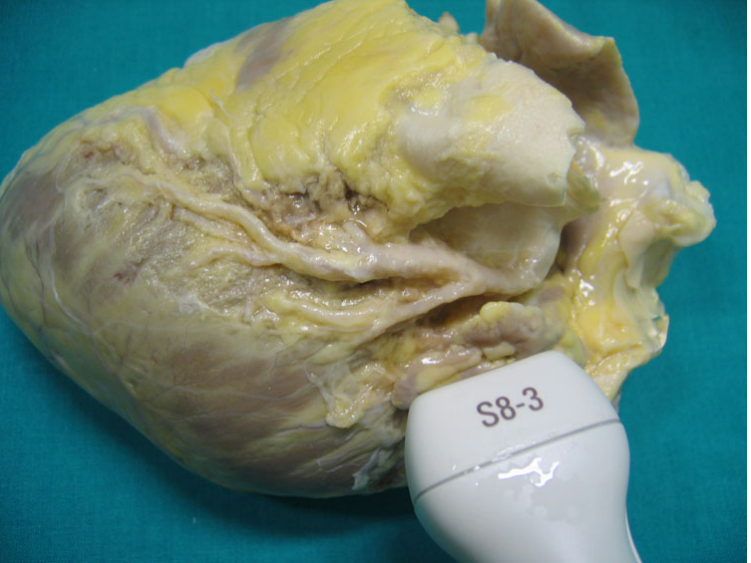
represents the anatomical findings of Intermediate tract of Left anterior descending coronary artery (reference keys are: the anterior interventricular Sulcus and Septal Branches).

**Figure 7:**
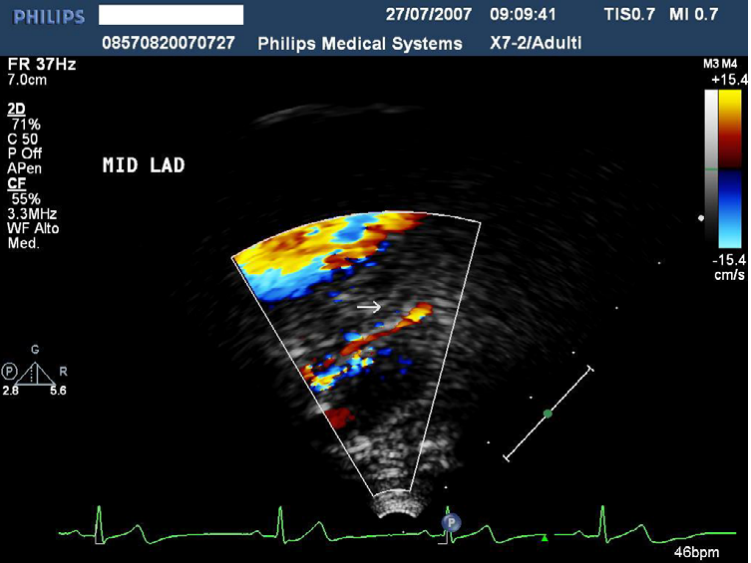
shows the ultrasound findings of proximal left anterior highlighted with color-Doppler.

**Figure 8:**
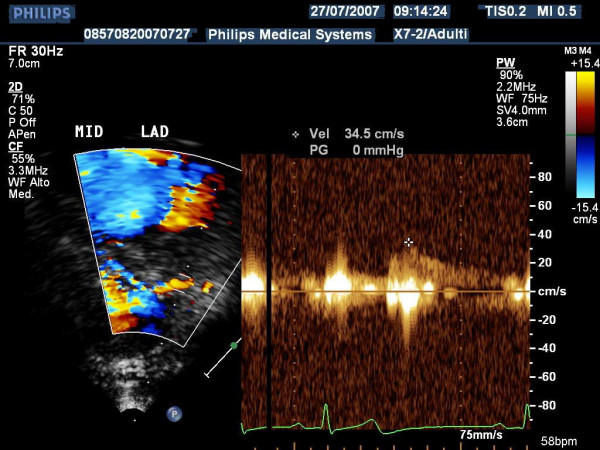
shows the Pulse Doppler envelope of mid tract of left descending coronary artery.

Intermediate Lad: the septal perforans branches represent the key references obtainable by angulating the probe slightly lower (3,5-5 MHz in 2^nd^) and maintaining the focus on the anterior interventricular sulcus (Fig 6, 7, 8) [see Additional File 3].

**Figure 9:**
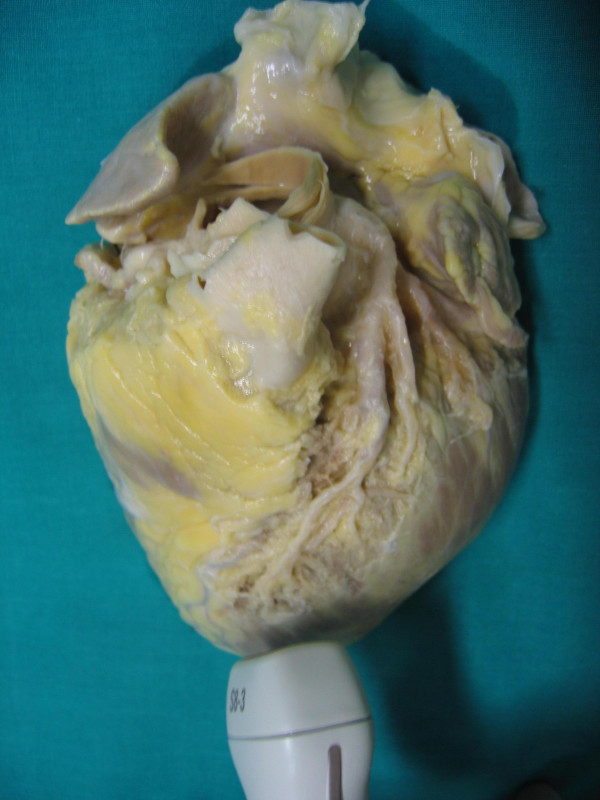
shows the anatomical finding of distal tract of Left anterior descending coronary art (the reference keys are: distal tract of the anterior interventricular Sulcus and Septal Branches).

**Figure 10:**
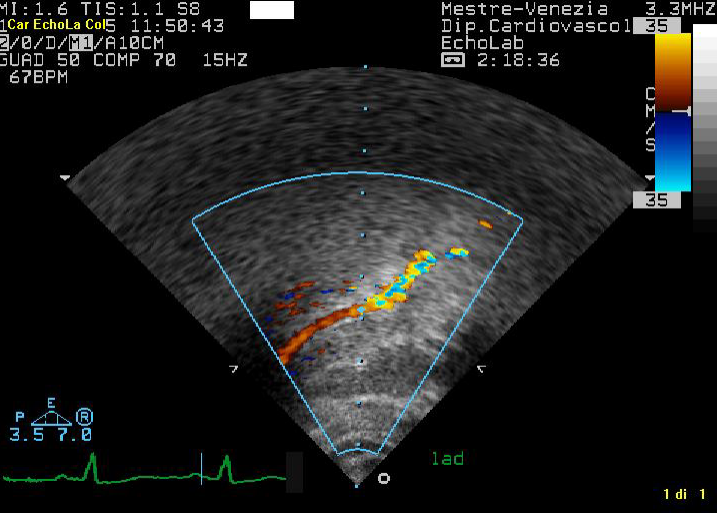
represents the Ultrasound findings of distal left anterior highlighted with color-Doppler.

**Figure 11:**
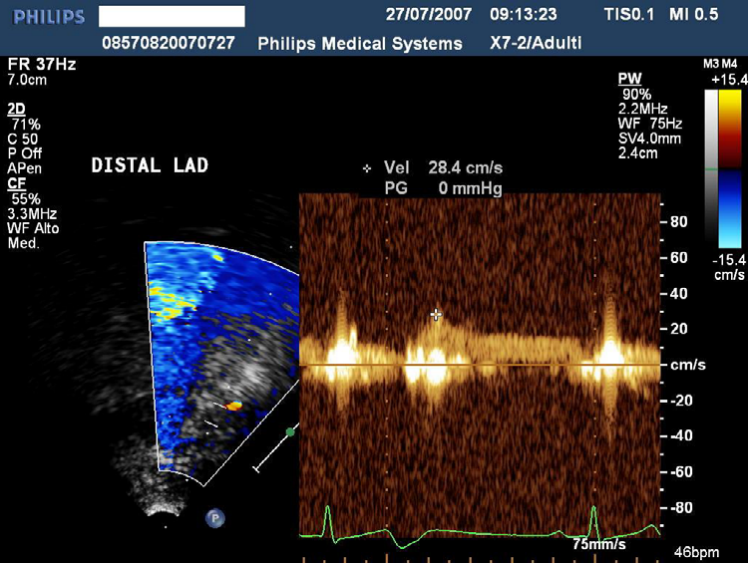
shows the Pulse Doppler envelope of distal tract of left descending coronary artery.

Distal Lad tract: this can be highlighted by investigating the lower part of interventricular anterior sulcus near the apex under Color Doppler guidance and adopting growing delivery frequencies (5–7 Mhz in 2^nd ^harmonic). By subsequently applying Pulse Doppler inside the coronary vessel, we may obtain the coronary spectrum and therefore quantify it (Fig 9, 10, 11) [see Additional File 4].

**Figure 12:**
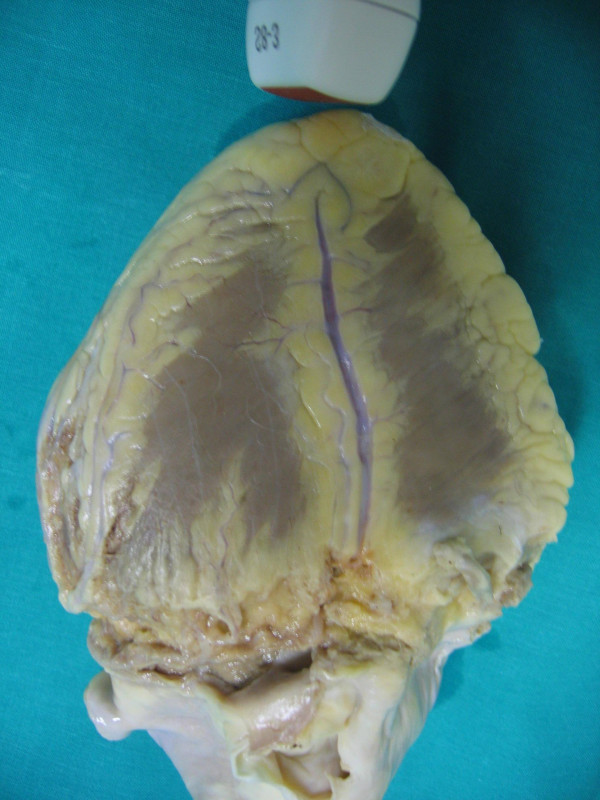
shows the anatomical findings of distal tract of Left posterior descending coronary artery (reference keys are: distal tract of the posterior interventricular Sulcus and Septal Branches).

**Figure 13:**
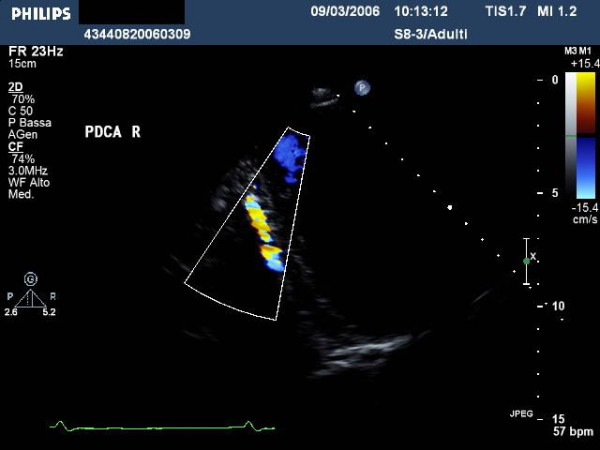
highlights the Ultrasound findings of distal left posterior descending coronary artery highlighted with Color-Doppler.

**Figure 14:**
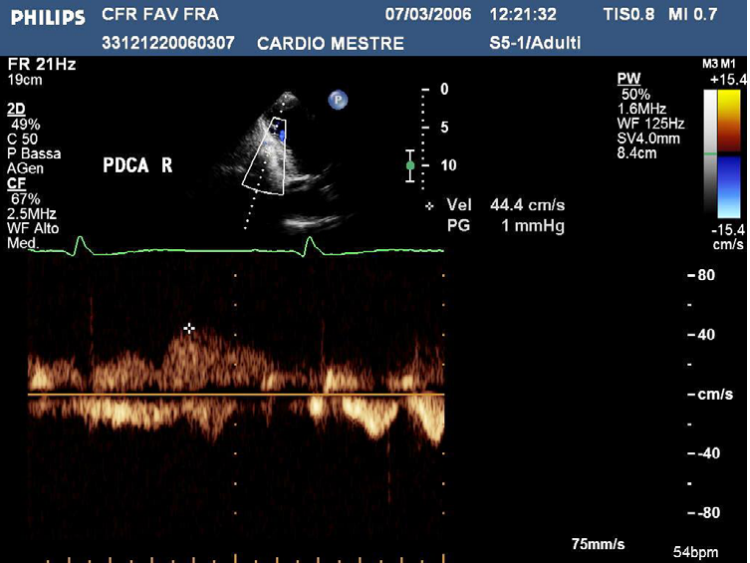
shows the Pulse Doppler envelope of the distal tract of left posterior coronary artery.

For the distal left posterior coronary artery we must address the ultrasound beam in a *counter-clockwise *direction (Fig 1b), and, always under Color Doppler guidance, focus the ultrasound beam on the posterior descending coronary sulcus (Fig. 12, 13, 14) [see Additional File 5].

By applying the latest technology, it is nowadays possible to investigate LAD in 98% of patients and PDCA in 60–70% of patients [1], obviously delivering appropriate ultrasound frequencies for each coronary target such as summarized in Tab 1.

## Tips and tricks: the importance of having a good setting

A good approach to the two major coronary arteries is made possible only by appropriate setting of the echo machine. To achieve this, it is important to apply the current parameters summarized in Table 1. A different application of the transducer exists for each coronary artery, ranging from lower frequencies in evaluating PDCA and proximal tract of LAD and higher frequencies for distal tract of LAD.

**Table 1:** Technical ultrasound application used to highlight the different coronary arteries

	Probe Delivery frequencies	Color Doppler PRF	Wall Filters	Pulse Doppler filters	Focus	Anatomical reference
LAD	4–8 MHz as 2^nd ^harmonic	15–25 cm/s	high	low	on	Anterior interventricular sulcus
PDCA	3.5–5 MHz as 2^nd ^harmonic	15–25 cm/s	high	low	on	Posterior interventricular sulcus

LAD = Left anterior descending coronary artery

PDca = Posterior descending coronary artery

To obtain correct coronary flow and reserve (CFR), we need to be sure where we address the sample volume and we should be able to maintain the same position during the entire injection of the vasodilator agent. We can use Dipyridamole (0,84 mg/Kg/m' over 6 m' continuously) as well as Adenosine (140 mcg/Kg/m' for 2–3 m' with infusion time depending on operator skills) as a hyperaemic stressor. Dipyridamole is preferable because it reaches the hyperaemic peak induction more slowly and therefore it allows us to simultaneously investigate left ventricle contractility and coronary flow in different tracts of the coronary artery and to obtain a flow reserve in both coronary arteries [1-3].

## Coronary pitfalls

Mistake could happen for several reasons, in particular:

For LAD:

- Mapping different coronary arteries from LAD such as Diagonal or the Intermediate branch

- Mapping different LAD tracts during the same investigation

- Misinterpreting wall noise, for example atrio-ventricular diastolic flow, as a coronary Doppler flow signal

- Misinterpreting epicardial space due to mild pericardial effusion as a coronary Doppler flow signal

-A lost coronary signal due to poor positioning of the probe during vasodilator infusion, a jumping of the investigation from one artery to another, the presence of pericardial effusion or right ventricular enlargement.

For PDCA:

- Misinterpreting wall noise, for example atrio-ventricular diastolic flow, as a coronary Doppler flow signal

- Investigating right ventricular flow, especially when this is enlarged

- Confusing the distal part of PDCA with the recurrent branch of the distal LAD tract that runs around the whole LV apex segment

- Not improving the coronary signal/noise ratio through the injection of a contrast agent

## Coronary ultrasound investigation – present and future

**Figure 15:**
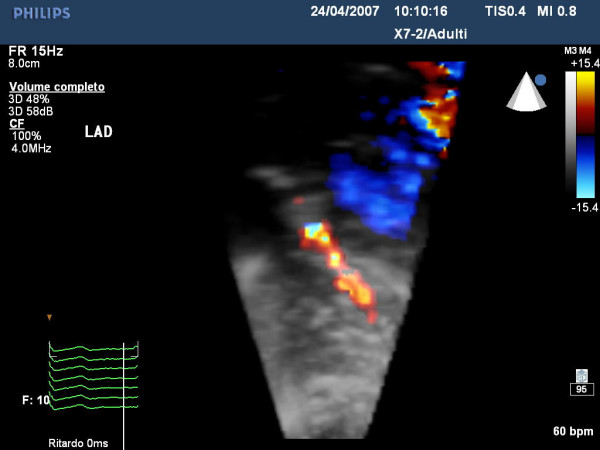
shows an example of the distal tract of left descending coronary artery highlights by 3-Dimensional Color Doppler evaluation.

A reasonable explanation why this coronary ultrasound approach has not become as widespread as expected lies in the fact that it requires good balance in terms of anatomical and technological knowledge. As a result, a dedicated learning curve is necessary, initially resulting in a significant loss of time. To convince a wider number of operators, we may well need to develop easier technology in terms of feasibility and application. Until recently, a good coronary signal could only be obtained by adopting different multi-frequency probes. Now, with the application of a broadband transducer we can obtain an excellent color flow coronary signal simply by switching different frequencies on a single probe. It is even possible to obtain a 3-dimensional view of coronary anatomy which allows us to focus coronary direction better and, therefore to guide coronary investigation better (Fig 15) [see Additional File 6]. The high quality of Color and Pulse Doppler obtainable with this transducer could facilitate the busy sonographer and guarantee a more objective coronary flow and reserve analysis.

## Supplementary Material

Additional file 1 Left anterior descending coronary artery. This video shows the Color Doppler of of left anterior descending coronary artery. Click here for file

Additional file 2 proximal tract of descending coronary artery. This video shows the Color-Doppler of proximal tract of descending coronary artery. Click here for file

Additional file 3 mid tract of descending coronary artery. This video shows the Color-Doppler of mid tract of descending coronary artery. Click here for file

Additional file 4 distal tract of descending coronary artery. This video shows the Color-Doppler of distal tract of descending coronary artery. Click here for file

Additional file 5 Mid-distal tract of descending posterior coronary artery. This video shows the Color-Doppler of mid-distal tract of descending posterior coronary artery. Click here for file

Additional file 6 3D Color-Doppler of distal tract of descending coronary artery. This video shows the 3D echo-Color_Doppler of distal tract of descending coronary artery as anatomical view. Click here for file
